# Safety and efficacy of short courses of antibiotic therapy in high-risk febrile neutropenic pediatric patients

**DOI:** 10.1038/s41375-026-02876-8

**Published:** 2026-02-04

**Authors:** Theresa Rohm, Konrad Bochennek, Isabel Taeuber, Jan-Henning Klusmann, Anke Barnbrock, Thomas Lehrnbecher

**Affiliations:** Department of Pediatrics, Division of Hematology, Oncology and Hemostaseology, Goethe University Frankfurt, Frankfurt am Main, Germany

**Keywords:** Medical research, Cancer

## To the Editor:

The majority of children and adolescents treated for acute leukemia experiences at least one infectious episode during neutropenia [1]. According to pediatric-specific guidelines, these patients are promptly hospitalized and receive empirical broad-spectrum antibiotics [2, 3], which further impairs the quality of life of patients and increases the risk of resistant pathogens [4]. Pediatric-specific guidelines strongly recommend for both high- and low-risk patients who have been clinically well, are afebrile for at least 24 h and have negative blood cultures at 48–72 h, to discontinue empirical antibiotic therapy if there is evidence of marrow recovery [3]. However, only in low-risk febrile neutropenic patients, a conditional recommendation was made for the discontinuation of antibiotics in this setting when there is no evidence for marrow recovery.

We expanded the recommendation to stop empirical antibiotic therapy irrespective of hematological recovery in our institution to high-risk patients defined by long periods of therapy-induced neutropenia (e.g., ≥10 days) [5, 6], such as patients with high-risk acute lymphoblastic leukemia (HR-ALL), acute myeloid leukemia (AML) or relapsed acute leukemia, and performed a retrospective audit to assess safety (e.g., mortality) and efficacy (e.g., readmission) of this approach.

This retrospective, single-center audit evaluated all consecutive patients <18 years treated for HR-ALL, AML or relapsed acute leukemia who had presented in stable condition with febrile neutropenia in order to assess outcome of antibiotic discontinuation and hospital discharge prior to hematological recovery. Patients were treated between April 2020 and March 2025 according to Berlin-Frankfurt-Münster (BFM)–based protocols, e.g., AIEOP-BFM-ALL2017 (EudraCT-2016-001935-12), AML-BFM-2017-Registry (DRKS 00013030) or INTREALL2010HR (EudraCT-2012-000810-12). Written informed consent for data collection and analysis was obtained within the consent procedure for cancer treatment and medical care approved by the local institutional review board (e.g., A105/18, 349/17, 16/0040). Clinical and microbiological data during intensive chemotherapy was extracted from the medical information system and entered in a secured database. The primary endpoint of the analysis was overall survival of the episode of infection. Secondary endpoints included readmission for suspected infection, or the days without antibiotic therapy from discharge until neutrophil recovery.

Prophylactic anti-infective measures for high-risk patients included systemic trimethoprim/sulfamethoxazole twice weekly and mold-active antifungal prophylaxis, but no systemic antibiotics [5, 7]. Diagnostics and first line therapy of febrile neutropenic patients in stable clinical condition consisted of piperacillin/tazobactam and tobramycin according to pediatric-specific guidelines [2, 3]. Tobramycin was discontinued in well-appearing patients after the first or second dosage. In AML patients, a glycopeptide was added to first line therapy because of the high risk of viridans-group streptococci. Antibiotic therapy was stopped and the patient was discharged if no pathogen was isolated in the bloodstream, the patient had received empirical intravenous antibiotics for at least 72 h, and was clinically well and afebrile for at least 48 h, irrespective of signs of hematological recovery. Patients with bloodstream infections were treated with antibiotics for 10-14 days, and were discharged without antibiotics prior to neutrophil recovery if they were in stable clinical condition, afebrile for ≥48 h and had a negative blood culture. Patients were then assessed at least every other day in the outpatient clinic, and blood counts were performed until hematological recovery.

Neutropenia was defined as an absolute neutrophil count (ANC) of <500/μl, fever as temperature >38.5 °C once or 38.0–38.5 °C twice within a 1 h interval [8]. Infectious episodes were categorized as fever of unknown origin (FUO; presence of fever without clinically or microbiologically identified focus), or as microbiologically or clinically documented infection [8]. A bloodstream infection was defined as a positive culture for pathogens isolated from peripheral blood or from the central venous catheter [8]. Diagnosis of an infection of the gastrointestinal tract required clinical symptoms and a plausible pathogen (e.g., *Clostridioides difficile*). Readmission was defined as hospital admission for a suspected infection (e.g., fever) within 7 days after discharge and prior to hematological recovery (ANC > 500/µl). First line antibiotic therapy, the strategy of antibiotic discontinuation and hospital discharge did not differ between first admission and readmission for a febrile neutropenic episode.

Statistical analysis was performed using SPSS 29.0 (IBM, USA). Comparisons were performed by *t* test, Wilcoxon signed-rank test, Mann–Whitney U test, and *Chi-*square test, as applicable. *P* values <0.05 were considered significant.

The median age (range) of the 25 boys and 12 girls was 8 years (0–17), and the underlying malignancies were HR-ALL (*n* = 12), relapsed ALL (*n* = 13), and AML (*n* = 12). Out of 156 infectious episodes assessed for eligibility, seven were excluded for unstable condition at presentation, direct transfer to the transplant unit, or death, respectively (Supplementary Figure).

Out of the analyzed 149 infectious episodes, the patient was discharged with an ANC < 500/µl in 114 episodes [ANC in 65 episodes (57%) <100/µl, in 49 episodes (43%) 100–500/µl] (Supplementary Figure). The median length of hospital stay (IQR) in these 114 episodes was 4 days (3–9), which corresponds to the number of days of antibiotics (Table 1). The median duration (IQR) of neutropenia of the episodes was 23 days (14-30). Fever of unknown origin was diagnosed in 95 episodes (83.3%), microbiologically and clinically documented infections in 18 (15.8%) and one (0.9%) episodes, respectively. Microbiologically documented infections consisted of eight bloodstream infections (two with Gram-positive, six with Gram-negative pathogens), six episodes of respiratory tract infections (all viral infections), and two episodes each with *Clostridioides difficile* diarrhea and bacterial soft tissue infection (Table 1).

**Table 1:** Characteristics of infectious episodes of pediatric high-risk patients discharged prior to hematological recovery.

	Total	With readmission	Without readmission	*P*
**Infectious episodes (n)**	**114**	**22**	**92**	
**Underlying malignancy**				
High-risk ALL, n (%)	53 (46.5)	9 (40.9)	44 (47.8)	
AML, n (%)	37 (32.4)	10 (45.5)	27 (29.3)	0.314
Relapsed ALL, n (%)	24 (21.1)	3 (13.6)	21 (22.8)	
**Duration of neutropenia [days; median (IQR)]**	**23 (14–30)**	**26 (23–33)**	**22 (13–29)**	**0.021**
**Days of antibiotic therapy [median (IQR)]**	**4 (3–9)**	**4 (3–6)**	**4 (3–9)**	**0.166**
**Hospital length of stay [days; median (IQR)]**	**4 (3–9)**	**4 (3–6)**	**5 (3–10)**	**0.166**
**FUO, n (%)**	**95 (83.3)**	**19 (86.4)**	**76 (82.6)**	**0.671**
**Microbiologically documented infection, n (%)**	**18 (15.8)**	**3 (13.6)**	**15 (16.3)**	**0.758**
Bloodstream infection, n (%)	8 (44.5)	1 (33.3)	7 (46.7)	
Gram-positive bloodstream infection, n	2	0	2	
	*Bacillus cereus* and *Cutibacterium acnes* (each *n* = 1)			
Gram-positive bloodstream infection, n	6	1	5	
	*Pseudomonas aeruginosa*, *Escherichia coli*, *Klebsiella* spp (each *n* = 2)			
Respiratory tract infection with isolation of plausible pathogen, n (%)*	6 (33.3)	1 (33.3)	5 (33.3)	
	Rhinovirus and Respiratory Syncycial Virus (each *n* = 2), Influenza-Virus and Bocavirus (each *n* = 1)			
Diarrhea with isolation of plausible pathogen, n (%)	2 (11.1)	1 (33.3)	1 (6.7)	
	*Clostridoides difficile* (n = 2)			
Soft tissue infection, n (%)	2 (11.1)	0	2 (13.3)	
	*Enterobacter cloacae* and *Pseudomonas aeruginosa* (each *n* = 1)			
**Clinically documented infection, n (%)**	**1 (0.9)**	**0**	**1 (1.1)**	**0.623**
Soft tissue infection				

*Note: patients with respiratory tract infections caused by a virus were handled identically compared to patients with FUO (e.g., initiation and discontinuation of antibiotics).

*ALL* acute lymphoblastic leukemia, *AML* acute myeloid leukemia, *IQR* interquartile range, *FUO* fever of unknown origin.

In 92 (80.7%) infectious episodes, follow-up until neutrophil recovery was without event, whereas in 22 episodes (19.3%), patients were readmitted neutropenic within 7 days of discharge. Nineteen patients had been discharged in the episodes of initial admission with the diagnosis of FUO, the other three had been treated for bloodstream infection (*Klebsiella* spp), RSV pneumonia and *C. difficile* diarrhea, respectively (Table 2). All except one of these patients were discharged with an ANC < 100/µl, one with an ANC of 150/µl. Median duration of neutropenia was significantly longer in patients with than in those without readmission (26 versus 22 days, *P* = 0.021). Readmission occurred in a median (IQR) of 2 days (2–3) after discharge. Upon readmission, seventeen patients (77.3%) presented with fever of 38.0–38.5 °C, and five (22.7%) with fever >38.5 °C; one patient exhibited hypotension as a potential sign of sepsis (Table 2). After readmission, microbiologically documented infections were diagnosed in five patients [two bloodstream infections (*Escherichia coli*, *Bacillus cereus;* both patients had been discharged in the episode of initial admission with FUO), two patients with RSV-infection (one persistent from initial admission, one newly diagnosed), and one patient with *Enterobacter cloacae* soft tissue infection]. The median length of hospital stay (IQR) upon readmission was 5 days (4–11). There was no death or ICU admission.

**Table 2:** Characteristics of infectious episodes of pediatric high-risk patients treated for febrile neutropenia and discharged prior to hematological recovery (“episode of initial admission”) and readmitted within 7 days with neutropenia and a suspected infection (“episode after readmission”).

Malignancy/age (years)	Category of infection	Days of antibiotics/in hospital	ANC at discharge (per µl)	Days from discharge to readmission	ANC at readmission (per µl)	ANC at discharge (per µl)	Fever > 38.5^o^C/ clinical problems	Category of infection	Days of antibiotics/in hospital
	Episode of initial admission				Episode after readmission				
Relapsed ALL/7	FUO	3/3	40	2	10	630	no/no	RSV-infection	9/9
HR-ALL/2	FUO	3/3	10	2	20	80	no/no	FUO	2/2
HR-ALL/2	FUO	3/3	0	3	0	70	no/no	FUO	6/6
AML/4	FUO	3/3	10	2	10	250	no/no	FUO	6/6
AML/4	FUO	3/3	10	2	0	320	no/no	FUO	12/12
HR-ALL/10	FUO	5/5	0	3	0	10	yes/no	FUO	4/4
AML/2	FUO	3/3	0	7	10	20	no/no	FUO	3/3
AML/17	FUO	4/4	0	3	0	40	no/no	BSI (*E. coli*)	17/17
AML/17	FUO	7/7	10	6	10	80	yes/no	BSI (*Bacillus cereus*)	7/7*
AML/17	BSI (*Klebsiella* spp)	14/14	0	3	10	50	yes/no	FUO	4/4
AML/17	FUO	5/5	10	1	10	30	no/no	FUO	13/13
AML/17	FUO	4/4	44	5	110	130	no/no	FUO	4/4
HR-ALL/10	Diarrhea *(C.diff)*	10/10	10	1	40	180	no/no	FUO	4/4
AML/13	FUO	8/8	10	4	10	50	yes/hypotension	Soft tissue *(E. cloacae*)	56/56
AML/17	FUO	5/5	0	1	70	880	no/no	Soft tissue (clinically)	18/18
Relapsed ALL/11	FUO	6/6	10	2	120	1390	yes/no	FUO	4/4
HR-ALL/8	FUO	3/3	10	2	0	10	no/no	FUO	4/4
HR-ALL/8	FUO	4/4	0	3	90	170	no/no	FUO	3/3
HR-ALL/8	FUO	3/3	10	3	0	20	no/no	FUO	3/3
Relapsed ALL/5	RSV-infection	9/9	10	2	270	450	no/no	RSV-infection	4/4
HR-ALL/2	FUO	4/4	10	2	10	250	no/no	FUO	7/7
HR-ALL/2	FUO	3/4	150	2	30	40	no/no	FUO	12/12

*for unknown reasons this episode was not treated according to internal standards.

*ALL* acute lymphoblastic leukemia. *HR* high-risk, *AML* acute myeloid leukemia, *FUO* fever of unknown origin, *BSI* bloodstream infection; *C. diff Clostridioides difficile*, *RSV* respiratory syncytial virus, *ANC* absolute neutrophil count.

Compared to a strategy where antibiotics are not discontinued and patients are not discharged prior to hematological recovery, our approach significantly reduced the median number of days of antibiotics and hospitalization (4 versus 14, and 5 versus 14, respectively; *P* < 0.001 each; analysis of 92 patients discharged with neutropenia and no readmission during follow-up) (data not shown).

As broad-spectrum antibiotics are associated with a number of negative effects, such as drug-drug interactions, the development of antimicrobial resistance, and an increased risk of invasive fungal and *Clostridioides difficile* infection, optimizing antibiotic duration is an important challenge [4, 9, 10]. In contrast to low-risk patients, pediatric data on safety and efficacy of short antibiotic courses in high-risk febrile neutropenic patients are scarce, which explains the fact that recent pediatric-specific guidelines have identified this question as a knowledge gap and do not give recommendations [2, 3]. Although several randomized trials in adult high-risk patients suggest the safety of this approach [11, 12], data have to be interpreted with caution as there are important differences between children and adults [13].

In patients with a bloodstream infection, adult guidelines recommend a minimum duration of 7 days of antibiotics with 4 days being afebrile [14], whereas no recommendation on the duration of antibiotics is given in the pediatric setting [2]. Notably, a recent randomized study did not find a difference in safety between 7 or 14 days of antibiotic therapy for Enterobacterales bacteremia in high‑risk neutropenic adults with 46% and 26% of patients being still neutropenic at discontinuation of antibiotics, respectively [15]. Again, transferring this data to the pediatric setting should be done with caution.

To our knowledge, our real-life data represent the largest case series of consecutive patients in this setting. Our analysis suggests that the recommendation of early stopping antibiotics irrespective of ANC in children at low-risk might safely be expanded to high-risk pediatric patients with FUO. Similarly, discontinuation of antibiotics irrespective of hematological recovery in children with bloodstream infection seems to be safe after a minimum treatment of ten days, as long as these children are afebrile and in good clinical condition. We recognize that our analysis does not replace sufficiently powered randomized studies, which would allow evidence-based pediatric specific recommendations. It is important to note that in all neutropenic children with early discontinuation of antibiotics, careful follow up and the ability to quickly return to the hospital in case of complications is a prerequisite, which might be a limitation of our strategy in individual institutions.

Our findings have some important implications. First, we significantly reduced with our strategy antibiotic exposure, which most likely will positively impact on adverse events and emergence of resistance [4]. Second, our strategy results in a significant reduction of hospitalization. This not only improves the quality of life of patients, but also significantly reduces bed occupancy and health care resources. Our data may be the basis for prospective randomized studies in high-risk pediatric patients, which ultimately lead to safely reducing antibiotic treatment, saving resources and improving the patients´ quality of life.

## Supplementary information

Supplementary information

## Data Availability

All data and material related to this study are available from the authors on request.
