# Supplementary material for: Safety and efficacy of short courses of antibiotic therapy in high-risk febrile neutropenic pediatric patients
Source: Leukemia. 2026 Feb 4;40(3):676–80. doi: 10.1038/s41375-026-02876-8 (PMC12960234; doi:10.1038/s41375-026-02876-8)
Supplement: Supplementary file 1 — Supplementary information [file 41375_2026_2876_MOESM1_ESM.docx]

**
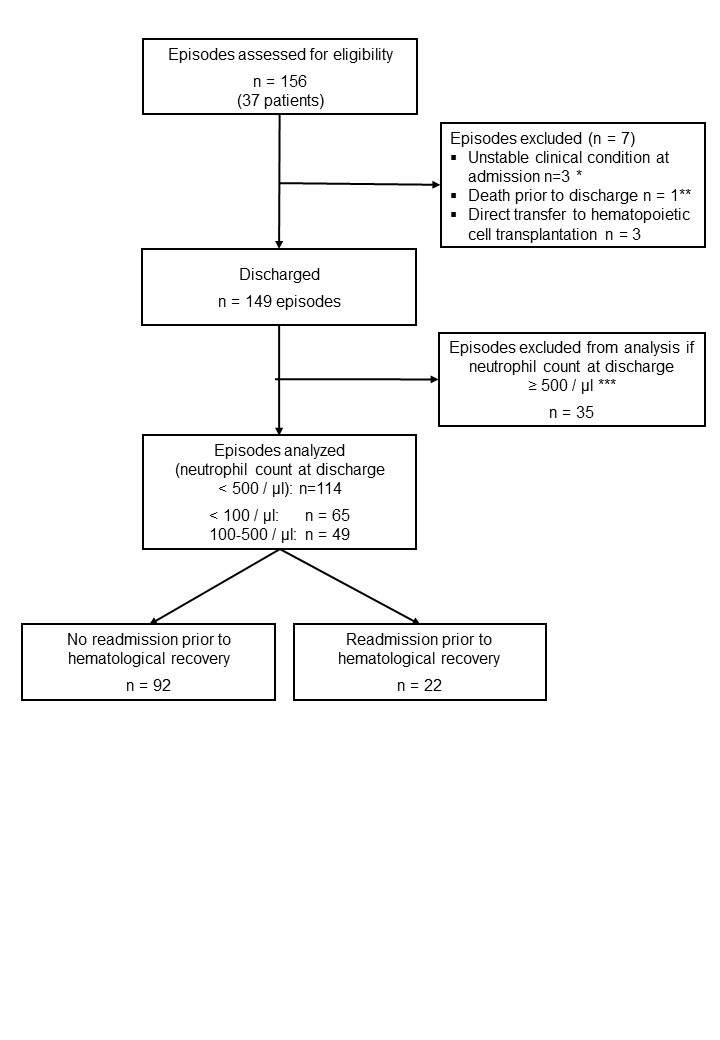
**

**Supplementary Figure**: Consort diagram of febrile neutropenic episodes in pediatric high-risk patients (high-risk ALL, AML, relapsed ALL) treated between April 2020 and March 2025

ALL acute lymphoblastic leukemia; AML acute myeloid leukemia

* Two patients with catheter related-sepsis, one with meningitis and altered mental status

** Death due to *Pseudomonas aeruginosa* sepsis

*** Discharge after hematological recovery was due to at least one of the following reasons: persistent fever (n=10), mucositis requiring pain management (n=6), poor clinical condition (n=3), antibiotic therapy of a microbiologically confirmed infection (n=12), and immediate continuation of chemotherapy after treatment of the infectious episode (n=15)
